# Prevalence and risk factors of ischemic monocular vision loss and concurrent brain ischemia

**DOI:** 10.1177/23969873231191577

**Published:** 2023-08-18

**Authors:** Cathy E Smith, Juraj Kukolja

**Affiliations:** 1 Department of Neurology and Clinical Neurophysiology, Helios University Hospital Wuppertal, Wuppertal, Germany; 2 Faculty of Health, Witten/Herdecke University, Witten, Germany

**Keywords:** Silent brain ischemia, retinal artery occlusion, cardiovascular risk factors

## Abstract

**Introduction:**

We performed a retrospective cohort study to identify predictors of concurrent asymptomatic brain ischemia in patients with ischemic monocular vision loss.

**Patients and methods:**

An inpatient database research of admissions to the Helios University Hospital Wuppertal, Germany between 01/2016 and 12/2020 was conducted. Inclusion criteria were confirmed diagnosis of transient monocular vision loss (MVL), retinal artery occlusion (RAO), and magnetic resonance imaging (MRI) of the brain within 10 days of MVL. Silent brain ischemia (SBI) was defined as diffusion restrictions with corresponding reduced apparent diffusion coefficient in MRI and an absence of neurological deficits besides those complying with MVL in clinical examination. The prevalence and cardiovascular predictors of SBI were analyzed with logistic regression and an artificial neural network.

**Results:**

One hundred fourteen out of 475 patients treated with monocular vision loss were included in this study. The mean age was 67.7 ± 13.6 years. 48.2% were male and 47.4% had RAO. MRI scan of the brain was performed after 3.9 ± 2.3 days and detected SBI in 17%. Age ⩾67 years, cardiac etiology of MVL, and cerebral ischemia in medical history were revealed as predictors of SBI in MRI.

**Conclusions:**

Patients older than 66 years, with a suspected cardiac embolism as the cause of RAO and previous cerebral ischemia, are more likely to present SBI in cerebral MRI. Therefore, MR imaging, particularly in these patients, can be useful.

## Introduction

Retinal artery occlusion (RAO) is one of the major causes of acute, painless visual loss. It has an incidence rate of 1–10 per 100,000 US citizens per year, increasing drastically with age and peaking at 80 years.^1,2^ RAO refers to permanent visual loss, while amaurosis fugax is a transient monocular vision loss (TMVL) and typically lasts less than 15 min but rarely can persist for up to 24  h.^3–6^ RAO can affect the central retinal artery (CRAO) or a branch retinal artery (BRAO).

Most RAO is of non-arteritic origin and often derives from large-artery atherosclerosis (LAA) of the ipsilateral internal carotid artery (ICA) or a cardiac source of embolism (up to 48%).^7–9^ In patients under 40 years of age, rare causes, such as thrombophilia, or vasculitis, should be ruled out.^3,7,9^ Giant cell arteritis (about 4% of RAO cases) should be considered in patients above 50 years, especially in TMVL patients or bilateral involvement.^3^

The most often associated cardiovascular risk factor with ischemic MVL is hypertension.^9–11^ Diagnostic workup identifies at least one new vascular risk factor in more than 60% of patients, in individuals with or without previous risk factors in medical history.^10,12^

Although ischemic MVL patients have an increased risk of cerebral ischemia, especially in the first 7 days, only every second or third patient is referred for a neurological evaluation.^13,14^ While covert strokes may be detected due to neurological symptoms and signs, silent brain ischemia (SBI) may be overlooked due to its asymptomatic nature. SBI is the most common incidental finding on cerebral MRI and presents most often as lacunar or cortical lesions with a diameter <1 cm in non-eloquent locations.^15–19^ Studies on ischemic MVL and concurrent SBI have shown a prevalence of up to 37%.^19–27^ As SBI seems to precede manifest infarction and doubles the risk of vascular dementia, its importance may not be underestimated.^28^

This study aimed to analyze the prevalence of asymptomatic cerebral ischemia in patients with ischemic MVL and detect an associated vascular risk profile to identify specific criteria that justify a cerebral MRI scan. Special consideration of risk constellations may improve the diagnostic workup of patients with ischemic MVL in the rising importance of economic-based treatment of inpatients.

## Methods

### Study design and study group

**Figure 1.**
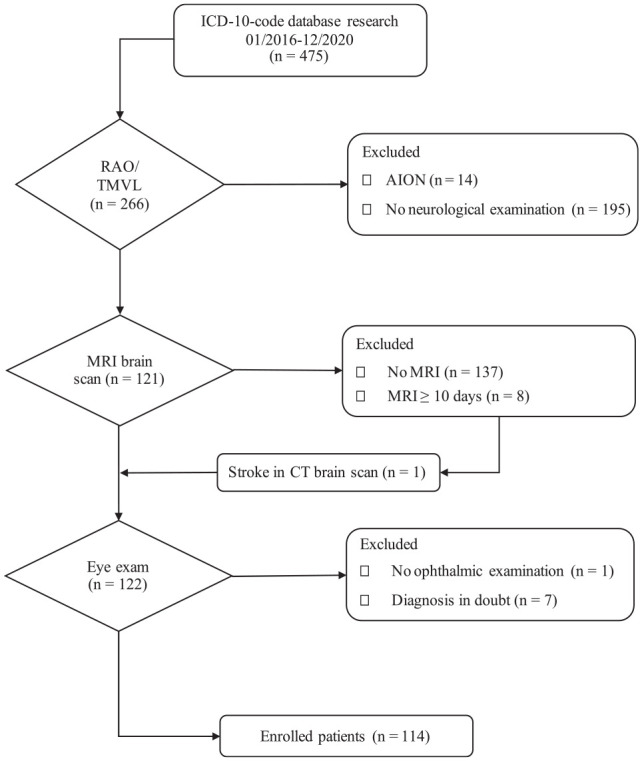
Patient enrollment procedure. Patient recruitment was based on patient database research and ICD-10-GM-codes. Fourteen wrongfully encoded patients with AION were identified via diagnosis confirmation via release letters. Only patients with a cerebral MRI scan within ten days of symptom onset were considered, except for one patient who already presented an ischemic stroke in a CT scan. AION: anterior ischemic optic neuropathy; CF: clinical features; CT: computed tomography; ICD: International Statistical Classification of Diseases and Related Health Problems; MRI: magnetic resonance imaging; RAO: retinal artery occlusion; TMVL: transient monocular vision loss.

This retrospective cohort study included inpatients 18 years of age or older treated at the Helios University Hospital Wuppertal, Germany, from January 2016 to December 2020. Patients were recruited per patient database research with the German Modification of the International Statistical Classification of Diseases and Related Health codes. Specific codes were used: G45.3 (amaurosis fugax), H34.0 (transient retinal artery occlusion), H34.1 (central retinal artery occlusion), H34.2 (other retinal artery occlusion), H34.9 (unspecified retinal artery occlusion). Furthermore, unspecific codes H53.8 (other visual disturbances) and H53.9 (unspecified visual disturbances) were added to the search to ensure the detection of all potential patients with transient ischemic vision loss and RAO despite unspecific coding. The diagnosis was confirmed in each case by using the release letter. The detailed enrollment process is shown in Figure 1.

### Cerebral imaging

All patients had a computed tomography scan of the head at presentation in our Department of Emergency Medicine. A cerebral MRI scan was performed within 10 days of symptom onset. SBI was classified as diffusion restriction in diffusion-weighted imaging (DWI) with corresponding reduced apparent diffusion coefficient (ADC) maps in patients with no history of neurological deficits besides MVL symptoms in the past 14 days. Lesions were correlated in fluid-attenuated inversion recovery (FLAIR) imaging as hyperintensities. MRI was performed with a 1.5-Tesla system by the Department of Radiology in the same hospital. Written reports regarding SBI, and its locations, were evaluated.

### Demographics, medical history, and etiologic classification

We analyzed baseline demographic data comprising age, sex, time to admission, and time to MRI scan. In addition, cardiovascular comorbidities (hypertension, diabetes mellitus, dyslipidemia, smoking status, atrial fibrillation, coronary artery disease, and previous strokes) were analyzed.

The etiology of ischemic MVL was assessed with the updated ASCOD-phenotyping classification: atherothrombosis, small-vessel disease, cardiac embolism, other causes, and dissection without further assessment of the grade of causality.^29^ All of the patients provided written consent for the utilization of their medical data for research purposes.

### Statistical analysis

All statistical analyses were performed with IBM SPSS Statistics software for Mac (V 28.0, SPSS, Inc. Chicago, IL, USA). Since we expected a comparable etiology of RAO and ischemic TMVL, both groups were pooled in order to obtain statistically more reliable results. We considered the combined risk profile to be more applicable to the clinical setting than separate analyses. Study group characteristics are presented as mean ± standard deviation (SD) or frequency (%). For comparison with published studies, SD was estimated from the interquartile range using the formula when necessary.^30^

$$\mathrm{SD}=\;\frac{3\mathrm{rd}\;\mathrm{quartile}\;-\;1\mathrm{st}\;\mathrm{quartile}}{2\;x\;0.6745}$$

Nominal variables were analyzed with Pearson’s chi-squared test or Fisher’s exact test, respectively, while continuous variables were analyzed with the student’s *t*-test or the Mann-Whitney-*U*-test according to assumptions to compare subgroups’ baseline demographic and clinical features.^31–33^ Multivariate binary logistic regression analysis was used to identify predictors for SBI. Nagelkerkes *R*^2^ was used to evaluate the fit of the model to the data.

As artificial neural networks are often more suitable for complex matters an alternative model was fitted with a multilayer perceptron (MLP) model which consists of an input layer with the independent predictor variables, one hidden layer and an output layer with the variable SBI.^34^ Batch training mode was used for model training and scaled conjugate gradient training was chosen as the optimization algorithm. Backpropagation was applied to minimize error after multiple iterations and cross-validation was applied to limit model overfitting.^32,34–36^ The hyperbolic tangent function was used as activation function in the input layer, whereas softmax was set as the activation function in the output layer. Cross-entropy was the error function. For more details on the MLP, Supplemental Material is available.

Test accuracy was evaluated by the area under the curve (AUC) and was compared between logistic regression and MLP.^32,37^ In this study, a *p*-value <0.05 was considered statistically significant. There were no missing values regarding model calculations.

### Ethics approval/statement

The approval of the Ethics Committee at the Faculty of Health of Witten/Herdecke University, Germany, was obtained to ensure this study complies with the guidelines of the Declaration of Helsinki in their latest version (World Medical Association, 2013). This manuscript adheres to the guidelines of the Strengthening the Reporting of Observational Studies in Epidemiology (STROBE) statement.^38^

## Results

A study group of 114 subjects was obtained. The mean age of all enrolled subjects was 67.7 ± 13.6 years (age range 18–90 years), and 48.2% were male (*n* = 55). The mean time to admission to our hospital was 0.8 days (0–7 days) in all patients. 38.6% were diagnosed with CRAO (*n* = 44), 8.8% had BRAO (*n* = 10), and 52.6% were treated with TMVL (*n* = 60). All visual impairments were unilateral (100%, *N* = 114), and 54.4% presented right-sided symptoms (*n* = 62). Baseline demographics, values of the cardiovascular risk profile and etiology of MVL of the study group and its RAO/TMVL and SBI/no-SBI subgroups, respectively, are presented in Table 1.

**Table 1.** Baseline demographics and clinical features.

Factor	RAO (*n* = 54)/TMVL (*n* = 60)	SBI (19)/no-SBI (95)	Total (*N* = 114)
Age (years)	71.3 ± 10.3/64.4 ± 15.3; (*p* = 0.017)	74.9 ± 8.8/66.2 ± 13.9; (*p* = 0.012)	67.7 ± 13.6
Sex (female)	27 (50.0)/32 (53.3); (*p* = 0.722)	12 (63.2)/47 (49.5); (*p* = 0.276)	59 (51.8)
Affected eye (right side)	34 (63.0)/28 (46.7); (*p* = 0.081)	13 (68.4)/49 (51.6) (*p* = 0.178)	62 (54.4)
ASCOD phenotyping	(*p* = 0.237)	(*p* < 0.001)	
Atherothrombosis	31 (57.4)/37 (61.7)	4 (21.1)/64 (67.4)	68 (59.6)
Small-vessel disease	7 (13.0)/13 (21.7)	2 (10.5)/18 (18.9)	20 (17.5)
Cardiac embolism	15 (27.8)/9 (15.0)	13 (68.4)/11 (11.6)	24 (21.1)
Other	1 (1.9)/-	-/1 (1.1)	1 (0.9)
Dissection	-/1 (1.7)	-/1 (1.1)	1 (0.9)
Cardiovascular risk factors			
Coronary artery disease	10 (18.5)/2 (3.3); (*p* = 0.012)	3 (15.8)/9 (9.5); (*p* = 0.419)	12 (10.5)
Previous stroke	7 (13.0)/4 (6.7); (*p* = 0.345)	5 (26.3%)/6 (6.3%); (*p* = 0.007)	11 (9.6)
Smoking (current and ex)	14 (25.9)/19 (31.7); (*p* = 0.757)	1 (5.2)/24 (25.3); (*p* = 0.146)	33 (28.9)
Atrial fibrillation	3 (5.6)/3 (5.0); (*p* = 1.0)	2 (10.5)/4 (4.2); (*p* = 1.0)	6 (5.3)
Hypertension	53 (98.1)/40 (66.7); (***p*** < 0.001)	18 (94.7)/75 (78.9); (*p* = 0.191)	93 (81.6)
Obesity	15 (28.3)^a^/12 (20.3)^b^; (*p* = 0.325)	4 (21.1)/23 (24.2); (*p* = 1.0)	27 (23.7)^c^
Dyslipidemia	29 (53.7)/24 (40.0); (*p* = 0.143)	11 (57.9)/42 (44.2); (*p* = 0.275)	53 (46.5)
Diabetes mellitus	6 (11.1)/8 (13.3); (*p* = 0.718)	3 (15.8)/11 (11.6); (*p* = 0.701)	14 (12.3)
Diagnosis of ⩾1 new risk factor(s)	11 (20.4)/16 (26.7); (*p* = 0.600)	1 (5.3)/26 (27.4); (*p* = 0.038)	27 (23.7)
Hypertension	1 (1.9)/11 (18.3); (*p* = 0.005)	1 (5.3)/11 (11.6); (*p* = 0.687	12 (10.5)
Diabetes mellitus	0^e^/4^f^ (7.4); (*p* = 0.051)	0/4^g^ (4.5); (*p* = 1.0)	4^h^ (3.7)
Dyslypidemia	5^e^ (8.3)/9^f^ (16.7); (*p* = 0.369)	0^i^/14^k^ (15.6); (*p* = 0.073)	14^h^ (3.0)
Atrial fibrillation	2 (3.4)/2 (3.8); (*p* = 1.0)	1 (5.3)/3 (3.2); (*p* = 0.523)	6 (5.3)
MRI after onset of symptoms (d)	4.4 ± 2.5^a,*^/3.5 ± 2.1; (*p* = 0.077)	4.9 ± 2.3/3.7 ± 2.3; (*p* = 0.033)	3.9 ± 2.3^d,*^
SBI in MRI	14 (25.9)/5 (8.3); (*p* = 0.012)	-	19 (16.7)

AF: amaurosis fugax; ASCOD: A: atherothrombosis, S: small-vessel disease, C: cardiac embolism, O: other causes, D: dissection; BMI: body mass index; d: days; MRI: magnetic resonance imaging; RAO: retinal artery occlusion; SD: standard deviation.

Continuous variables are presented as mean ± SD, and categorical values are presented as frequency (%). Obesity is defined as BMI ⩾ 30 kg/m^2^.

a *n* = 53.

b *n* = 59.

c *n* = 112.

d *n* = 113.

e *n* = 56.

f *n* = 52.

g *n* = 89.

h *n* = 108.

I *n* = 18.

k *n* = 90.

* One patient without MRI due to asymptomatic cerebral ischemia in CT scan.

About 16.7% (*n* = 19) proved to have SBI in the MRI, whereas 57.9% of SBI were detected in the territory supplied by the middle cerebral artery (*n* = 11), followed by 15.8% of posterior cerebral artery strokes (*n* = 3), and 10.5% of anterior cerebral artery strokes (*n* = 2). SBI in multiple territories was detected in 15.8% (*n* = 3).

The distinction of etiology in the SBI versus no-SBI group comparison was highly significant (*p* < 0.001). Primarily, a cardiac embolism was associated with SBI (SBI: 68.4%; no SBI: 11.6%). Previous cerebral ischemia prevalence was significantly higher in subjects with SBI (26.3%) than without SBI (6.3%; *p* = 0.007), while other medical preconditions were not associated with SBI.

A multivariate logistic regression model (*n* = 114; *p* < 0.001) with the predictor variables age ⩾67 years (*p* = 0.010, OR = 9.178, 95% CI, 1.697–49.642), prior stroke (*p* = 0.005, OR = 11.560, 95% CI, 2.131–62.696), and cardiac etiology (*p* < 0.001, OR = 22.985, 95% CI, 5.825–90.698) was fitted after backward elimination with all prior determined significant predictor variables. Nagelkerkes *R*^2^ was 0.507 which showed a moderate fit of the model to the data. The sensitivity of SBI prediction in this model was 63.2%, the specificity 93.7%, the classification accuracy 88.6%, and the positive predictive value 66.7%. AUC was 0.899 (95% CI, 0.830–0.968).

**Figure 2.**
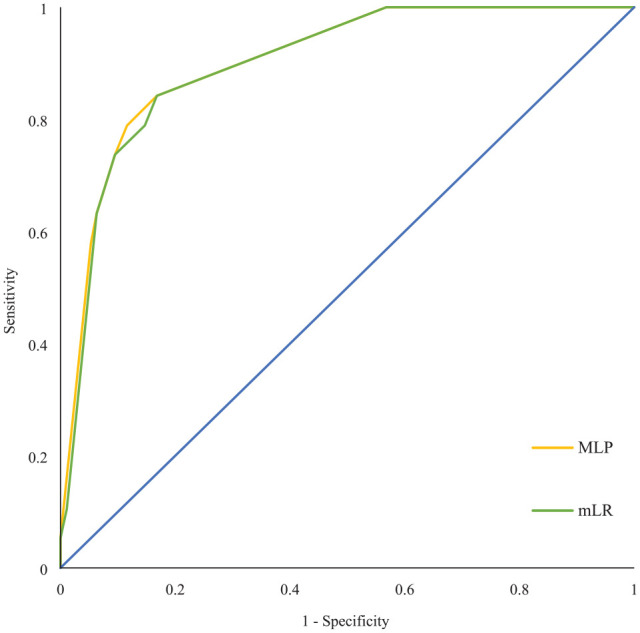
ROC curves for MLP and LR models. This figure shows the ROC curve of the MLP model with the combined training and testing sample, and mLR model. AUC of the MLP for predicted SBI was 0.904 (95% CI 0.835–0.972), and AUC of mLR was 0.899 (95% CI 0.830–0.968). Both multivariable models show a reasonable degree of selectivity. Independent parameters were age ⩾67 years, cardiac source, and previous cerebral ischemia. AUC: area under the curve; MLP: multilayer perceptron; mLR: multivariate logistic regression; ROC: receiver operating characteristics curve; SBI: silent brain infarction.

A multilayer perceptron neural network with the same prediction parameters was fitted as an alternative model. All 114 patients were divided into three samples. Seventy-two patients were assigned to the training sample (63.2%), 29 to the testing sample (25.4%), and 13 to the holdout sample (11.4%). The sensitivity of the combined training and test sample was 52.9%, while the specificity was 89.7%, and the test accuracy was 86.8%. The area under the curve (AUC) for SBI in MRI was 0.904 (95% CI, 0.835–0.972). Figure 2 shows the receiver operating curves (ROC) of the multivariate logistic regression model and the MLP.

## Discussion

In the present study, the prevalence of SBI in patients with MVL was 17% which corresponds to prior studies reporting a prevalence of SBI in RAO between 15% and 21%^19–21,25,26^ and to the pooled SBI rate of 18% reported in the meta-analysis by Fallico et al.^39^ (see Table 2). SBI was found in different locations, which complied with the observations made by Zhang et al.^20^ Higher or lower prevalence of asymptomatic brain ischemia reported in some studies was most likely due to differing inclusion or exclusion criteria, definitions of SBI diagnosis and baseline demographics.^22–24,27^

**Table 2.** Prevalence of SBI and predictors in different studies. Comparison of conducted study results on monocular visual loss and silent brain infarct in MRI with the results of this study.

Study	Study period	Study group (*N*)	TMVL (%)	Age (y)	SBI prevalence (%)	MRI interval (d)
This study	2016–2020	114	53	68 ± 14	17	⩽10
Golsari et al.^19^	Unknown	112	35	69 ± 12^§^	15	⩽7
Zhang et al.^20^	2013–2016	41	56	65 ± 14^§^	20	⩽7
Kim et al.^21^	2003–2018	244	0	65 ± 15	18	⩽14
Lee et al.^22^	2005–2012	33	0	58 ± 17	9	⩽7
Helenius et al.^24^	2000–2008	129	57	64 ± 16	11,*	⩽7
Lavin et al.^23^	2009–2017	103	0	65 ± 13	37.5^†^	-
Ayrignac et al.^25^	2015–2016	103	44	72 ± 12^§^	20	⩽7
Lauda et al.^26^	2008–2013	213	32	75 ± 8^§^	21^‡^	⩽7
Laczynski et al.^27^	2004–2018	221	0	66 ± 15	<2	-

D: days; DWI: diffusion-weighted imaging; ICA: internal carotid artery; LAA: large-artery atherosclerosis; MRI: magnetic resonance imaging; RAO: retinal artery occlusion; SBI: silent brain infarction; SD: standard deviation; TMVL: transient monocular vision loss; y: years.

* Approximate value of prevalence of SBI by study details: 17 of the 31 DWI lesion-positive patients presented hemispheric symptoms.^24^

† Neurological evaluation was not sought.^23^

‡ Approximate value of prevalence of SBI of patients without additional neurological symptoms.^26^

§ Calculated SD as outlined in Methods: Statistical analysis.^30^

A multivariate logistic regression model identified the parameters age ⩾67, cardiac source of embolism, and previous stroke in medical history as predictors of SBI in MRI. The MLP network yielded comparable statistical values (see Results). Concerning clinical practicability, sensitivity, specificity, and PPV, the multivariate logistic regression model was the best of the presented models.

Another major finding of the present study is that the likelihood of SBI increases by 6.5% with each year of age. The age-dependent occurrence of SBI is supported by the findings of Kim et al.^21^ and Lauda et al.^26^ and follows the general understanding that stroke is age-related.^40^ We showed a sixfold greater risk of SBI in MRI for patients older than 67 years, indicating that this age group primarily benefits from an adjunctive MRI in the diagnostic evaluation process. We were also able to prove that patients with TMVL are four times less likely to present SBI in cerebral MRI than patients with RAO, which is confirmed by Lauda et al.,^26^ who found significantly more SBI in MRI in patients with CRAO or BRAO. Another main finding of our study was the importance of a cardiac embolic source as a predictor of SBI, although LAA was the most frequent cause of MVL (60%). Regarding the cardiovascular risk profile, hypertension was the most common (82%) and the most often newly diagnosed risk factor in our study group, especially in TMVL (18%). This corroborates previous findings reporting hypertension as the most frequent risk factor for MVL.^10,12^ Diagnostic workup led to a diagnosis of new risk factors in 24% of cases, highlighting its importance for secondary prevention. Last, our statistical analysis showed that MVL patients with ischemic stroke in medical history were more likely to have SBI in MRI.

A limitation of this study was the retrospective nature, leading to a selection bias and patients not meeting the inclusion criteria. The imbalanced study group size of patients with SBI (*n* = 19) and no-SBI (*n* = 95) complicated the statistical prediction of the SBI risk profile.

In conclusion, especially patients with an ischemic MVL who are 67 years old or older and present with a positive history of cerebral ischemia and suspected cardiac embolic source might benefit from MRI performance. Since an MRI brain scan may be beneficial in patients with undetermined MVL etiology additionally, the indication should be generous to detect underlying pathomechanisms. However, a prospective study has yet to prove the sufficiency of these predictors. Importantly, our study confirms that RAO patients require a diagnostic workup comparable to an overt stroke to prevent further ischemic events. Since acute RAO is a rare disease, immediate referral to a specialized center such as a hospital with a department of ophthalmology and a stroke unit should be advised for diagnostic workup and appropriate individual therapeutic decisions, for example (currently off-label) intravenous or intraarterial thrombolysis which showed promising results on improving visual impairment.^41,42^

## Supplemental Material

sj-doc-2-eso-10.1177_23969873231191577 – Supplemental material for Prevalence and risk factors of ischemic monocular vision loss and concurrent brain ischemia Click here for additional data file. Supplemental material, sj-doc-2-eso-10.1177_23969873231191577 for Prevalence and risk factors of ischemic monocular vision loss and concurrent brain ischemia by Cathy E Smith and Juraj Kukolja in European Stroke Journal

sj-docx-1-eso-10.1177_23969873231191577 – Supplemental material for Prevalence and risk factors of ischemic monocular vision loss and concurrent brain ischemia Click here for additional data file. Supplemental material, sj-docx-1-eso-10.1177_23969873231191577 for Prevalence and risk factors of ischemic monocular vision loss and concurrent brain ischemia by Cathy E Smith and Juraj Kukolja in European Stroke Journal
