# Supplementary material for: Prevalence and risk factors of ischemic monocular vision loss and concurrent brain ischemia
Source: Eur Stroke J. 2023 Aug 18;8(4):982–8. doi: 10.1177/23969873231191577 (PMC10683730; doi:10.1177/23969873231191577)
Supplement: sj-doc-2-eso-10.1177_23969873231191577 – Supplemental material for Prevalence and risk factors of ischemic monocular vision loss and concurrent brain ischemia [file sj-doc-2-eso-10.1177_23969873231191577.doc]

STROBE Statement—Checklist of items that should be included in reports of ***cohort studies***

|  | Item No | Recommendation |
| --- | --- | --- |
| **Title and abstract** | 1 | *(*a) Indicate the study’s design with a commonly used term in the title or the abstract  Page 1  *“We performed a retrospective cohort study to identify predictors of concurrent asymptomatic brain ischemia in patients with ischemic monocular vision loss.”* |
|  |  | (*b*) Provide in the abstract an informative and balanced summary of what was done and what was found  Page 1-2  *“Methods: An inpatient database research of admissions to the Helios University Hospital Wuppertal, Germany between 01/2016 and 12/2020 was conducted. Inclusion criteria were confirmed diagnosis of transient monocular vision loss (MVL), retinal artery occlusion (RAO) and magnetic resonance imaging (MRI) of the brain within ten days of MVL. Silent brain ischemia (SBI) was defined as diffusion restrictions with corresponding reduced apparent diffusion coefficient in MRI and an absence of neurological deficits besides those complying with MVL in clinical examination. The prevalence and cardiovascular predictors of SBI were analyzed with logistic regression.*  *Results: 114 out of 475 patients treated with monocular vision loss were included in this study. The mean age was 67.7 ± 13.6 years. 48.2% were male, and 47.4% had RAO. MRI scan of the brain was performed after 3.9 ± 2.3 days and detected SBI in 17%. Multivariate logistic regression and an artificial neural network revealed age ≥ 67 years, cardiac etiology of MVL, and cerebral ischemia in medical history as predictors of SBI in MRI.”* |
| Introduction | | |
| Background/rationale | 2 | Explain the scientific background and rationale for the investigation being reported  Page 3  *“Studies on ischemic MVL and concurrent asymptomatic stroke have shown a prevalence of up to 37%. 27-35 A silent brain infarction (SBI) is a diffusion-restricted cerebral lesion in magnetic resonance imaging (MRI) without any clinical correlation. 36,37 SBI is the most common incidental finding on cerebral MRI and presents most often as lacunar or cortical lesions with a diameter < 1 cm in non-eloquent locations. 27,36,38-40 As SBI seems to precede manifest infarction and doubles the risk of vascular dementia, its importance may not be underestimated. 41”* |
| Objectives | 3 | State specific objectives, including any prespecified hypotheses  Page 3-4  *“This study aimed to analyze the prevalence of asymptomatic cerebral ischemia in patients with ischemic MVL and detect an associated vascular risk profile to identify specific criteria that justify a cerebral MRI scan. Special consideration of risk constellations may improve the diagnostic workup of patients with ischemic MVL in the rising importance of economic-based treatment of inpatients.”* |
| Methods | | |
| Study design | 4 | Present key elements of study design early in the paper  Page 5  *“This retrospective cohort study included inpatients 18 years of age or older treated at the Helios University Hospital Wuppertal, Germany, from January 2016 to December 2020. Patients were recruited per patient database research with the German Modification of the International Statistical Classification of Diseases and Related Health codes. Specific codes were used: G45.3 (amaurosis fugax), H34.0 (transient retinal artery occlusion), H34.1 (central retinal artery occlusion), H34.2 (other retinal artery occlusion), H34.9 (unspecified retinal artery occlusion). Furthermore, unspecific codes H53.8 (other visual disturbances) and H53.9 (unspecified visual disturbances) were added to the search to ensure the detection of all potential patients with transient ischemic vision loss and RAO despite unspecific coding. The diagnosis was confirmed in each case by using the release letter. The detailed enrollment process is shown in Figure 1”* |
| Setting | 5 | Describe the setting, locations, and relevant dates, including periods of recruitment, exposure, follow-up, and data collection.  Page 5  *“This retrospective cohort study included inpatients 18 years of age or older treated at the Helios University Hospital Wuppertal, Germany, from January 2016 to December 2020. Patients were recruited per patient database research with the German Modification of the International Statistical Classification of Diseases and Related Health codes. Specific codes were used: G45.3 (amaurosis fugax), H34.0 (transient retinal artery occlusion), H34.1 (central retinal artery occlusion), H34.2 (other retinal artery occlusion), H34.9 (unspecified retinal artery occlusion). Furthermore, unspecific codes H53.8 (other visual disturbances) and H53.9 (unspecified visual disturbances) were added to the search to ensure the detection of all potential patients with transient ischemic vision loss and RAO despite unspecific coding. The diagnosis was confirmed in each case by using the release letter. The detailed enrollment process is shown in Figure 1”* |
| Participants | 6 | *(*a) Give the eligibility criteria, and the sources and methods of selection of participants. Describe methods of follow-up  See Figure 1 |
|  |  | (*b*) For matched studies, give matching criteria and number of exposed and unexposed  Not applicable |
| Variables | 7 | Clearly define all outcomes, exposures, predictors, potential confounders, and effect modifiers. Give diagnostic criteria, if applicable  Page 7  *“SBI was classified as diffusion restriction in diffusion-weighted imaging (DWI) with corresponding reduced apparent diffusion coefficient (ADC) maps in patients with no history of neurological deficits besides MVL symptoms in the past 14 days. Lesions were correlated in fluid-attenuated inversion recovery (FLAIR) imaging as hyperintensities.”* |
| Data sources/ measurement | 8* | For each variable of interest, give sources of data and details of methods of assessment (measurement). Describe comparability of assessment methods if there is more than one group  Pages 7  *“We analyzed baseline demographic data comprising age, sex, time to admission, and time to MRI scan. In addition, cardiovascular comorbidities (hypertension, diabetes mellitus, dyslipidemia, smoking status, atrial fibrillation, coronary artery disease, and previous strokes) were analyzed.*  *The etiology of ischemic MVL was assessed with the updated ASCOD-phenotyping classification: atherothrombosis, small-vessel disease, cardiac embolism, other causes, and dissection without further assessment of the grade of causality. 29 All of the patients provided written consent for the utilization of their medical data for research purposes.”* |
| Bias | 9 | Describe any efforts to address potential sources of bias  See Legend of Figure 1  *“14 wrongfully encoded patients with AION were identified via diagnosis confirmation via release letters.”* |
| Study size | 10 | Explain how the study size was arrived at  See Figure 1 |
| Quantitative variables | 11 | Explain how quantitative variables were handled in the analyses. If applicable, describe which groupings were chosen and why  Page 8  *“Study group characteristics are presented as mean ± standard deviation (SD) or frequency (%). For comparison with published studies, SD was estimated from the interquartile range…”* |
| Statistical methods | 12 | (*a*) Describe all statistical methods, including those used to control for confounding  Pages 8-9  *“As artificial neural networks are often more suitable for complex matters an alternative model was fitted with a multilayer perceptron (MLP) model which consists of an input layer with the independent predictor variables, one hidden layer and an output layer with the variable SBI. 34 Batch training mode was used for model training and scaled conjugate gradient training was chosen as the optimization algorithm. Backpropagation was applied to minimize error after multiple iterations and cross-validation was applied to limit model overfitting. 32, 34-36 The hyperbolic tangent function was used as activation function in the input layer, whereas softmax was set as the activation function in the output layer. Cross-entropy was the error function. For more details on the MLP, supplemental material is available.*  *Test accuracy was evaluated by the area under the curve (AUC) and was compared between logistic regression and MLP. 32, 37 In this study, a p-value < .05 was considered statistically significant. There were no missing values regarding model calculations.”* |
|  |  | (*b*) Describe any methods used to examine subgroups and interactions  Page 8  *“Nominal variables were analyzed with Pearson’s chi-squared test or Fisher’s exact test, respectively, while continuous variables were analyzed with the student’s t-test or the Mann-Whitney-U-test according to assumptions to compare subgroups’ baseline demographic and clinical features. Nagelkerkes R^2^ was used to evaluate the fit of the model to the data.”* |
|  |  | (*c*) Explain how missing data were addressed  Page 9  *“There were no missing values regarding model calculations.”* |
|  |  | (*d*) If applicable, explain how loss to follow-up was addressed  Not applicable |
|  |  | (*e*) Describe any sensitivity analyses  Page 9  *“In this study, a p-value < .05 was considered statistically significant.”* |
| Results | | |
| Participants | 13* | (a) Report numbers of individuals at each stage of study—eg numbers potentially eligible, examined for eligibility, confirmed eligible, included in the study, completing follow-up, and analysed  See Figure 1 |
|  |  | (b) Give reasons for non-participation at each stage  See Figure 1 |
|  |  | (c) Consider use of a flow diagram  Figure 1 |
| Descriptive data | 14* | (a) Give characteristics of study participants (eg demographic, clinical, social) and information on exposures and potential confounders  Page 10  *“A study group of 114 subjects was obtained. The mean age of all enrolled subjects was 67.7 ± 13.6 years (age range 18 – 90 years), and 48.2% were male (n = 55). The mean time to admission to our hospital was 0.8 days (0 – 7 days) in all patients. 38.6% were diagnosed with CRAO (n = 44), 8.8% had BRAO (n =10), and 52.6% were treated with TMVL (n = 60). All visual impairments were unilateral (100%, N = 114), and 54.4% presented right-sided symptoms (n = 62). Visual disturbances lasted in 61.7% of the TMVL group (n= 37) less than 15 minutes, as reported by the patients. Baseline demographics, values of the cardiovascular risk profile and etiology of MVL of the study group and its RAO/ TMVL and SBI/ no-SBI subgroups, respectively, are presented in Table 1.”* |
|  |  | (b) Indicate number of participants with missing data for each variable of interest  See Table 1 |
|  |  | (c) Summarise follow-up time (eg, average and total amount)  No follow up |
| Outcome data | 15* | Report numbers of outcome events or summary measures over time |
| Main results | 16 | (*a*) Give unadjusted estimates and, if applicable, confounder-adjusted estimates and their precision (eg, 95% confidence interval). Make clear which confounders were adjusted for and why they were included  Page 13  *“A multivariate logistic regression model (n=114; p < .001) with the predictor variables age ≥ 67 years (p = .010, OR = 9.178, 95% CI, 1.697 – 49.642), prior stroke (p = .005, OR = 11.560, 95% CI, 2.131 – 62.696), and cardiac etiology (p < .001, OR = 22.985, 95% CI, 5.825 – 90.698) was fitted after backward elimination with all prior determined significant predictor variables. Nagelkerkes R^2^ was 0.507 which showed a moderate fit of the model to the data. The sensitivity of SBI prediction in this model was 63.2%, the specificity 93.7%, the classification accuracy 88.6%, and the positive predictive value 66.7%. AUC was 0,899 (95% CI, 0.830 – 0,968).* |
|  |  | (*b*) Report category boundaries when continuous variables were categorized  Not applicable |
|  |  | (*c*) If relevant, consider translating estimates of relative risk into absolute risk for a meaningful time period  Not applicable |
| Other analyses | 17 | Report other analyses done—eg analyses of subgroups and interactions, and sensitivity analyses  Page 12  *“16.7% (n = 19) proved to have SBI in the MRI, whereas 57.9% of SBI were detected in the territory supplied by the middle cerebral artery (n = 11), followed by 15.8% of posterior cerebral artery strokes (n = 3) and 10.5% of anterior cerebral artery strokes (n = 2). SBI in multiple territories was detected in 15.8% (n = 3).* |
| Discussion | | |
| Key results | 18 | Summarise key results with reference to study objectives  Page 15  *“In the present study, the prevalence of SBI in patients with MVL was 17% which corresponds to prior studies reporting a prevalence of SBI in RAO between 15 and 21% 19-21, 25, 26 and to the pooled SBI rate of 18% reported in the meta-analysis by Fallico, Lotery 39 (see Table 2). SBI was found in different locations, which complied with the observations made by Zhang, Zhang 20 Higher or lower prevalence of asymptomatic brain ischemia reported in some studies was most likely due to differing inclusion or exclusion criteria, definitions of SBI diagnosis and baseline demographics. 22-24, 27*  *A multivariate logistic regression model identified the parameters age ≥ 67, cardiac source of embolism, and previous stroke in medical history as predictors of SBI in MRI. The MLP network yielded comparable statistical values (see Results). Concerning clinical practicability, sensitivity, specificity, and PPV, the multivariate logistic regression model was the best of the presented models.* |
| Limitations | 19 | Discuss limitations of the study, taking into account sources of potential bias or imprecision. Discuss both direction and magnitude of any potential bias  Page 18  *“A limitation of this study was the retrospective nature, leading to a selection bias and patients not meeting the inclusion criteria. The imbalanced study group size of patients with SBI (n=19) and no-SBI (n= 95) complicated the statistical prediction of the SBI risk profile.”* |
| Interpretation | 20 | Give a cautious overall interpretation of results considering objectives, limitations, multiplicity of analyses, results from similar studies, and other relevant evidence  Pages 15-17  *“Another major finding of the present study is that the likelihood of SBI increases by 6.5% with each year of age. The age-dependent occurrence of SBI is supported by the findings of Kim, et al. 29 and Lauda, et al. 34 and follows the general understanding that stroke is age-related. 55 We showed a 6-fold greater risk of SBI in MRI for patients older than 67 years, indicating that this age group primarily benefits from an adjunctive MRI in the diagnostic evaluation process. We were also able to prove that patients with TMVL are four times less likely to present SBI in cerebral MRI than patients with RAO, which is confirmed by Lauda, et al. 34, who found significantly more SBI in MRI in patients with CRAO or BRAO. Another main finding of our study was the importance of a cardiac embolic source as a predictor of SBI, although LAA was the most frequent cause of MVL (60%). Regarding the cardiovascular risk profile, hypertension was the most common (82%) and the most often newly diagnosed risk factor in our study group, especially in TMVL (18%). This corroborates previous findings reporting hypertension as the most frequent risk factor for MVL. 14,16 Diagnostic workup led to a diagnosis of new risk factors in 24% of cases, highlighting its importance for secondary prevention. Last, our statistical analysis showed that MVL patients with ischemic stroke in medical history were more likely to have SBI in MRI.”* |
| Generalisability | 21 | Discuss the generalisability (external validity) of the study results  Page 18  *“Importantly, our study confirms that RAO patients require a diagnostic workup comparable to an overt stroke to prevent further ischemic events. Since acute RAO is a rare disease, immediate referral to a specialized center such as a hospital with a department of ophthalmology and a stroke unit should be advised for diagnostic workup and appropriate individual therapeutic decisions, e. g. (currently off-label) intravenous or intraarterial thrombolysis which showed promising results on improving visual impairment. 41, 42”* |
| Other information | | |
| Funding | 22 | Give the source of funding and the role of the funders for the present study and, if applicable, for the original study on which the present article is based  Page 19  None |

*Give information separately for exposed and unexposed groups.

**Note:** An Explanation and Elaboration article discusses each checklist item and gives methodological background and published examples of transparent reporting. The STROBE checklist is best used in conjunction with this article (freely available on the Web sites of PLoS Medicine at http://www.plosmedicine.org/, Annals of Internal Medicine at http://www.annals.org/, and Epidemiology at http://www.epidem.com/). Information on the STROBE Initiative is available at http://www.strobe-statement.org.
