# Supplementary material for: Prevalence and risk factors of ischemic monocular vision loss and concurrent brain ischemia
Source: Eur Stroke J. 2023 Aug 18;8(4):982–8. doi: 10.1177/23969873231191577 (PMC10683730; doi:10.1177/23969873231191577)
Supplement: sj-docx-1-eso-10.1177_23969873231191577 – Supplemental material for Prevalence and risk factors of ischemic monocular vision loss and concurrent brain ischemia [file sj-docx-1-eso-10.1177_23969873231191577.docx]

**Supplemental Material for the manuscript “Prevalence and risk factors of ischemic monocular vision loss and concurrent brain ischemia”**

The multivariate logistic regression model used in this study proved to be a moderately good fit to the data. We used a multilayer perceptron (MLP) model to assess whether a better model than the multivariate logistic regression could be yielded.

The MLP model is a unidirectional feedforward artificial neural network that copies neural principles of transmission.

In our study, we fitted a three-layer model consisting of an input layer, the hidden layer and an output layer. Each layer has nodes which are connected by a weight calculated by the MLP. The independent parameters “Age <67/ ≥ 67”, “no cardiac embolism/ cardiac embolism” and “no previous stroke/ previous stroke” are represented by six nodes in the input layer. The hidden layer contains three hidden nodes, while the output layer has two neurons, “SBI/ no SBI” (see Figure 1). Nodes are equivalent to neurons. Since categorical parameters were used, all variables were dummy encoded.

|  |
| --- |
| **Figure 1:** **Multilayer perceptron model (MLP)**  This figure shows the construction of the used MLP model and its three layers. Synaptic weights show the association between two connected neurons and are calculated from the training data but are not used for model interpretation. A bias is integrated into the input and hidden layer. H: Hidden neuron; SBI: Silent brain infarction. |

The MLP uses backpropagation as a training mode which processes deviance between predicted output data and actual data and reintroduces the knowledge to render calculated weights more precisely. Weights are comparable to regression coefficients. This minimizes the error after multiple iterations.

The input and hidden layer are activated by non-linear activation functions enabling the processing of complex questions. The input layer is activated by a hyperbolic tangent function that calculates the neurons of the hidden layer. A sigmoid function activates the hidden layer and relates to the output layer. A bias is integrated into the input and hidden layer.

Cross-validation was applied by assigning the study group to a training sample, testing sample, and holdout sample to limit model overfitting.

Batch training mode updates the weights after the training data has been processed completely.

We used scaled conjugate gradient training, a supervised learning algorithm as an optimization algorithm. Exact training settings are shown in Table 1.

| **Table 1: Scaled conjugate gradient training settings** | | |
| --- | --- | --- |
| Training parameters |  | Values |
| Initial Lambda |  | 0.0000005 |
| Initial Sigma |  | 0.000005 |
| Interval Center |  | 0 |
| Interval Offset |  | ± 0.5 |
| Set training options for the scaled conjugate gradient algorithm.  MLP: Multilayer perceptron. | | |

Cross-entropy was the error function, which quantifies the difference between expected and predicted parameter outcomes and updates and refines the weights. The cross-entropy error was 19,800. The cross-entropy error in the test sample was 8,102.

The normalized importance of the independent variables indicated the individual impact on the output variable; the higher the value, the greater the impact. The normalized importance of the individual parameters was 79.7% for age ≥ 67, 100% for cardiac etiology, and 91.6% for previous brain stroke.

Stopping rules were one consecutive step with no decrease in error, a maximum training time of 15 minutes, a minimum relative change in training error of 0.0001, and a minimum relative change in training error ratio of 0.001. The applied stopping rule in this study was one step without a decrease in error which occurred after four milliseconds.

**Reference(s):**

Backhaus, K, Erichson, B, Weiber, R. 5 Neuronale Netze, in Fortgeschrittene Multivariate Analysemethoden : Eine anwendungsorientierte Einführung. 2015; Springer Gabler: Berlin, Heidelberg. p. 295-347.

Traverso, A, Dankers, FJWM, Osong, B, Wee, L, van Kuijk, SMJ. Diving Deeper into Models, in Fundamentals of Clinical Data Science, P. Kubben, M. Dumontier, and A. Dekker, Editors. 2019; Springer International Publishing: Cham. p. 126-129.

Wee, L, van Kuijk, SMJ, Dankers, FJWM, Traverso, A, Welch, M, Dekker, A. Reporting Standards and Critical Appraisal of Prediction Models, in Fundamentals of Clinical Data Science, P. Kubben, M. Dumontier, and A. Dekker, Editors. 2019; Springer International Publishing: Cham. p. 135-150.
